# Changing Paradigms in the Initial Treatment of Ectopic Pregnancy at a University Hospital in Brazil

**DOI:** 10.1055/s-0043-1768999

**Published:** 2023-05-24

**Authors:** Bárbara Virginia Gonçalves Tavares, Letícia Sathler Delfino, Isabella Silvestre Ignarro, Luiz Francisco Baccaro

**Affiliations:** 1 Department of Obstetrics and Gynecology, Universidade Estadual de Campinas, Campinas, SP, Brazil

**Keywords:** Pregnancy complications, Pregnancy, Tubal, Pregnancy trimester first, Uterine hemorrhage, Complicações na gravidez, Gravidez tubárea, Gravidez primeiro trimester, Hemorragia uterina

## Abstract

**Objective** To evaluate the use of different treatment options for ectopic pregnancy and the frequency of severe complications in a university hospital.

**Methods** Observational study with women with ectopic pregnancy admitted at UNICAMP Womeńs Hospital, Brazil, between 01/01/2000 and 12/31/2017. The outcome variables were the type of treatment (first choice) and the presence of severe complications. Independent variables were clinical and sociodemographic data. Statistical analysis was carried out by the Cochran–Armitage test, chi-square test, Mann–Whitney test and multiple Cox regression.

**Results** In total 673 women were included in the study. The mean age was 29.0 years (± 6.1) and the mean gestational age was 7.7 (± 2.5). The frequency of surgical treatment decreased significantly over time (z = -4.69; p < 0.001). Conversely, there was a significant increase in the frequency of methotrexate treatment (z = 4.73; p < 0.001). Seventy-one women (10.5%) developed some type of severe complication. In the final statistical model, the prevalence of severe complications was higher in women who were diagnosed with a ruptured ectopic pregnancy at admission (PR = 2.97; 95%CI: 1.61–5.46), did not present with vaginal bleeding (PR = 2.45; 95%CI: 1.41–4.25), had never undergone laparotomy/laparoscopy (PR = 6.69; 95%CI: 1.62–27.53), had a non-tubal ectopic pregnancy (PR = 4.61; 95%CI: 1.98–10.74), and do not smoke (PR = 2.41; 95%CI: 1.08–5.36).

**Conclusion** there was a change in the first treatment option for cases of ectopic pregnancy in the hospital during the period of analysis. Factors inherent to a disease that is more difficult to treat are related to a higher frequency of severe complications.

## Resumo

**Objetivo** Avaliar as diferentes opções de tratamento para gravidez ectópica e a frequencia de complicações graves em um hospital universitário.

**Métodos** Estudo observacional com mulheres com gravidez ectópica admitidas no Hospital da Mulher da UNICAMP, no Brasill, entre 01/01/2000 e 31/12/2017. As variáveis de desfecho foram o tipo de tratamento (primeira escolha) e a presença de complicações graves. As variáveis independents foram dados clínicos e sociodemográficos. A análise estatística foi realizada pelo teste de Cochran–Armitage, teste de qui-quadrado, teste de Mann–Whitney e Regressão de Cox Múltipla.

**Resulados** No total, 673 mulheres foram incluídas no estudo. A idade médica foi de 29.0 anos (± 6.1) e a idade gestacional media foi de 7.7 (± 2.5). A frequencia de tratamento cirúrgico diminuiu significativamente ao longo dos anos(z = -4.69; p < 0.001). Simultaneamente, houve um aumento da frequencia do tratamento clínico(z = 4.73; p < 0.001). Setenta e uma mulheres (10.5%) desenvolveram algum tipo de complicação grave. No modelo estatístico final, a prevalência de complicações graves foi maior nas mulheres que tiveram diagnóstico de gestação ectópica rota à admissão (PR = 2.97; 95%CI: 1.61–5.46), que não apresentaram sangramento vaginal (PR = 2.45; 95%CI: 1.41–4.25), sem antecedentes de laparotomia/laparoscopia (PR = 6.69; 95%CI: 1.62–27.53), com gravidez ectópica não-tubária (PR = 4.61; 95%CI: 1.98–10.74), e não tabagistas (PR = 2.41; 95%CI: 1.08–5.36).

**Conclusão** Houve uma mudança na escolha do primeiro tratamento indicado nos casos de gravidez ectópica durante o período analisado. Os fatores inerentes a doença relacionados a maior dificuldade de tratamento foram associados a maior frequencia de complicações graves.

## Introduction

An ectopic pregnancy is one in which the blastocyst is implanted in a location other than the uterine cavity. In most cases of ectopic pregnancy, the fallopian tube is the most common site of implantation, although it can occur at other sites, such as the ovaries, uterine scar, intestinal loops, cervix, and uterine horn. 1 2 Ectopic pregnancy accounts for 2% of all pregnancies and is one of the main obstetric emergencies. 3 4 5 It is one oh the main causes of maternal death in the first trimester of pregnancy. This is an indicator of poor quality of health care services provided to women because most of those deaths are preventable. 6 7

Advances in early diagnosis can help in the adoption of less invasive treatments and reduce death rate. 8 Ectopic pregnancy can be treated with methotrexate, a folate antagonist, using different protocols. It can also be treated by different surgical techniques, such as laparotomy or laparoscopy. Tubal ectopic pregnancy is generally treated either by salpingostomy or salpingectomy. 8 9 Laparoscopy is currently considered the best approach for cases in which surgical intervention is indicated as well as non-tubal cases, that fulfill two preconditions: hemodynamically stable and the availability of a team of experienced laparoscopist. 3 8 10

What makes the difference in managing ectopic pregnancy is the ability to provide high-quality, cost-effective treatment that will yield maximum patient satisfaction. However, this might not be possible in some locations. In low- and middle-income countries where early diagnosis is not possible, careful selection of treatment is often difficult because most patients are usually brought to the hospital facility in emergencies. 11 12 Brazil, which is considered a middle-income country, has a relatively high maternal mortality rate, which is still far from the United Nation targets. Concomitantly, there is a scarcity of studies evaluating the morbidity and mortality associated with ectopic pregnancy as well as the treatments provided. 7 8 9 Considering the importance of this disease in the establishment of a fundamental index for women's health and the scarcity of data specific to Brazil, we sought to compare the rates of methotrexate, surgical, and expectant management in a university hospital in the south eastern region of the country. We also evaluated the laparoscopic rate and the frequency of severe complications.

## Methods

This was an observational study involving all women admitted at the University of Campinas (UNICAMP) Women's Hospital, Campinas, Brazil, between January 1, 2000 and December 31, 2017, who had a confirmed diagnosis of ectopic pregnancy, registered either at admission or at discharge. The University of Campinas (UNICAMP) Women's Hospital is a tertiary-level hospital, located in the south eastern region of Brazil. It usually receives cases of pregnancy-related complications from several cities in the region. The hospital handles an average of 250 deliveries and 20 first trimester pregnancy complications per month. Ectopic pregnancy cases were identified using the following International Classification of Diseases (ICD), 10th revision codes: O00 (ectopic pregnancy), O00.0 (abdominal pregnancy), O00.1 (tubal pregnancy), O00.2 (ovarian pregnancy), O00.8 (other ectopic pregnancy), and O00.9 (ectopic pregnancy, unspecified). Data was collected by the researchers in charge of the Medical Archive and Statistics Service of the hospital after careful analysis of the medical records. Data containing cases other than ectopic pregnancy were excluded from the study. The project was approved by the UNICAMP Research Ethics Committee (CAAE 53019116.6.0000.5404). It was a convenience sample. To calculate the power of the sample, the proportion estimate was used in a study descriptive with a categorical qualitative variable, in this case the estimate of surgical treatment of 64.34%, the clinical treatment estimate of 25.85%, the expectant treatment estimate of 9.81%, and an estimated presence of severe complications of 10.55% in a sample of n = 673 women, and setting the alpha significance level or type I error at 5% (alpha = 0.05) (or confidence interval of 95%) and the sampling error of 5% (d = 0.05). According to results, a power of 75.4% was obtained for surgical treatment, of 86.4% for clinical, 99.8% for expectant treatment and 99.8% for the presence of complications severe ( Table 1 ).

**Table 1:** Results of calculating the power of the sample to estimate the prevalence of the type of treatment of 1st choice and the presence of severe complications in women with ectopic pregnancies

Variables	n	Prevalence	Power of the sample	Sample Size for 80% power
Surgical treatment	n = 673	64,34%	0.754	n = 747
Methotrexate treatment	n = 673	25,85%	0.864	n = 543
Expectant management	n = 673	9,81%	0.998	n = 180
Presence of severe complications	n = 673	10.55%	0.998	n = 201

*Calculation of sample power considering proportion values of the current sample size, setting the alpha significance level at 5% (tipe I error) Calculation of sample size considering sample power at 80% (type II erro ror 20% beta), according to Hulley et al. (2007) 13 and Cohen (1988) 14

As this was a retrospective study based on database review, not compromising the privacy of subjects, the University of Campinas Research Ethics Committee waived the signing of informed consent. This article was prepared in accordance with the Strengthening the Reporting of Observational Studies in Epidemiology guidelines.

We considered as outcome variables: the type of treatment, administered as first choice after the diagnosis of ectopic pregnancy was made, which could be methotrexate, expectant or surgical management, the surgical approach (laparotomy or laparoscopy), and the presence of severe complications, defined as the presence of any of the following conditions during hospitalization: blood transfusion necessity, ICU (Intensive Unity Care) admission, surgical reassessment, hysterectomy, or death due to ectopic pregnancy.

We considered as independent variables: the year of occurrence of ectopic pregnancy, ectopic pregnancýs location, diameter of the gestational sac, serum quantitative β-hCG at diagnosis (measured in mUI/ml), fetal heartbeat on ultrasound, ectopic pregnancy integrity at diagnosis and during evolution, gestational age at diagnosis (calculated by the date of the last menstruation and by ultrasound analysis when available), woman's age, weight, height, body surface area, body mass index, skin color, schooling, marital status, parity, previous ectopic pregnancy history, history of tubal ligation, history of pelvic inflammatory disease, surgical history such as laparotomy or laparoscopy, history of intrauterine device as a contraceptive method as well as use during the diagnosis of ectopic pregnancy, symptoms reported when seeking emergency care—abdominal pain, vaginal bleeding, absence of symptoms, or other symptoms reported, smoking and current pregnancy resulting from in vitro fertilization, short or long methotrexate administration protocol, 13 and methotrexate dose administered. We defined the integrity of ectopic pregnancy at hospital presentation based on ultrasound and clinical features. In cases of unsuccessful methotrexate treatment, we evaluated the number of days from methotrexate treatment to the surgery and the reason for the indication of secondary treatment: pain, rupture of the gestational sac, and absence of biochemical response of β-hCG. In cases of methotrexate treatment was replaced by surgical treatment justified by the rupture of ectopic pregnancy during the follow-up, the rupture was defined by clinical and ultrasound findings. The methotrexate protocols used was short and long protocols. The short protocol consists of administering a dose of 50mg/m ^2^ of body surface and repeat the dose if β-hCG levels does not drop by at least 15% between days 4 and 7 after treatment. The long protocol consists of administering 1mg/kg/day in days 1, 3, 5 and 7, alternating with the administration of folinic acid in dose 0,1mg/kg/day – the protocol can e interrupted before 8 doses as long as β-hCG levels drops 15% or more between days. 1 Once the surgical approach was chosen as the initial treatment, we analyzed the: reason for the indication (described as absolute contraindication to methotrexate treatment, relative contraindication to methotrexate treatment, or option of medical staff), access route (Pfannenstiel laparotomy, median laparotomy, laparoscopy), type of surgery (salpingectomy, salpingophorectomy, salpingostomy), and the integrity of the contra lateral tube.

First, we performed a descriptive analysis of the data. Continuous variables were expressed as mean, standard deviation, median, minimum, and maximum. Categorical variables were expressed as relative frequencies. In order to compare the frequency of the types of treatment first indicated after the diagnosis of ectopic pregnancy, the surgical access route and the presence of serious complications between the years analyzed, the Cochran–Armitage test (trend test) was performed. Subsequently, bivariate analysis was performed to verify the association between the dependent variable “severe complication in cases of ectopic pregnancy” and the independent variables. For categorical independent variables, the chi-square test or Fisher's exact test was performed; for continuous variables, the Mann–Whitney test was performed. Multiple analysis by Cox regression was then performed. The level of significance was assumed to be 5%. The Statistical Analysis System for Windows version 9.2 (SAS Institute Inc., 2002-2008, Cary, NC, USA) was used.

## Results

During the evaluation period, the total number of cases identified with an ICD code of admission or discharge corresponding to ectopic pregnancy was 673. The mean age of women was 29.0 ± 6.1 years and the mean BMI was 25.44 (±4.9), including minimum BMI 16.23, q1 BMI 22.04; median BMI 24.46, q3 BMI 27.82, and maximum BMI 43.87. Three hundred and eighty-two women (73.5%) had a partner .and 70.1% were white. Of the patients evaluated: 23.9% were primiparous, 29.8% had undergone at least one cesarean section, 40.4% had previously had at least one abortion, 15.6% had previously had an ectopic pregnancy, 3.5% had undergone tubal ligation, and 23.3% had undergone laparotomy or laparoscopy. The main clinical and socioeconomic characteristics are described in Table 2 .

**Table 2:** The main clinical and socioeconomic characteristics

Characteristics	n	%
Age (y)		
< 20	39	5.79
20–29	308	45.77
30–39	303	45.02
40–49	23	3.42
Years of schooling*		
≤ 9	110	44.5
≤ 12	111	44.5
> 12 (college)	26	11.0
Marital status*		
With partner	382	73.5
Without partner	138	26.5
Skin color*		
White	420	70.1
Brown	129	21.6
Black	47	7.8
Yellow	2	0.3
Indigenous	1	0.2
Previous pregnancies*		
0	161	23.96
1	187	27.83
2	141	20.98
≥ 3	183	27.23
Previous cesarean sections*		
0	471	70.19
1	136	20.27
≥ 2	64	9.54
Previous abortions*		
0	400	59.52
1	187	27.83
≥ 2	85	12.65

The majority (94%) of the ectopic pregnancies were located in the fallopian tube. The mean gestational age was 7.4 ± 2.8 weeks when counted from the first day of the last menstrual period and 7.7 ± 2.5 weeks when determined by ultrasound. The mean diameter of the gestational sac was 37.2 ± 20.1 mm, and the mean serum β-hCG level was 5,783.2 ± 11,585.0. A visible fetal heartbeat was identified in 13.9% of cases, and 59.6% of the ectopic pregnancies were not ruptured at diagnosis. At admission, 87.0% of the women were symptomatic, 74% had abdominal pain, while 71.0% had vaginal bleeding. Twenty women (2.98%) had an ectopic pregnancy while using an intrauterine device, and seven (1.0%) had an ectopic pregnancy after an assisted reproduction procedure. Most of the treatments initially indicated, were surgical, (salpingectomy by Pfannenstiel laparotomy). When opting for methotrexate treatment, in the vast majority, a short protocol was performed with a mean methotrexate dose of 86.9 ± 22.1 mg ( Table 3 ).

**Table 3:** Treatments indicated for ectopic pregnancy (n = 673)

Treatments	Frequency (%)
Type of treatment (first choice)	
Surgical	64.64
Methotrexate	25.85
Expectant	9.81
Methotrexate protocol	
Short	98.32
Long	1.68
Surgery access route	
Pfannenstiel	64.24
Laparoscopy	21.22
Median incision	13.56
Laparoscopy followed by Pfannenstiel	0.98
Type of surgery	
Salpingectomy	77.57
Salpingophorectomy	4.30
Salpingostomy	8.01
Other	10.16

**Fig. 1:**
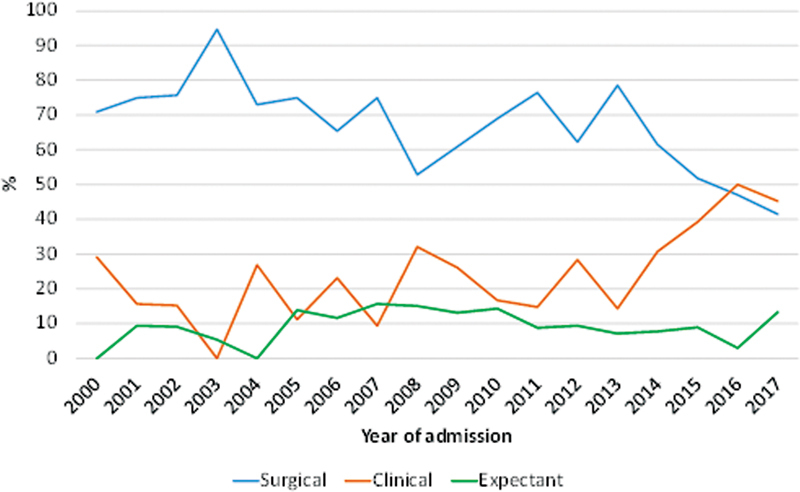
Type of treatment (first choice) between 2000 and 2017* Cochran-Armitage test: Clinical: z = 4.73; p < 0.001; Surgical: z = -4.69; p < 0.001; Expectant: z = 0.58; p = 0.561

The frequency of surgical treatment for ectopic pregnancy decreased significantly over time. In the year 2000, 70.9% of the women underwent surgical treatment, compared with only 41.5% in 2017 (z = -4.69; p < 0.001). Conversely, there was a significant increase in the frequency of methotrexate treatment, from 29.0% in 2000 to 45.2% in 2017 (z = 4.73; p < 0.001). As for expectant management, we did not observe a trend toward a change over time (z = 0.58; p = 0.561). These data are shown in detail in Figure 1 .

**Fig. 2:**
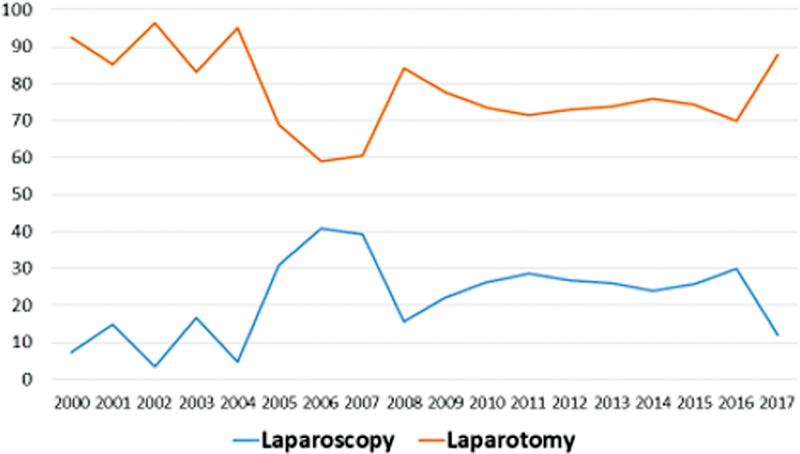
Surgical access routes between 2000 and 2017* Cochran-Armitage test: z = 2.09; p = 0.03

Once clinical methotrexate treatment was indicated, we have successful in 47.4% of the treatments. 33.5% need surgery after methotrexate treatment, and the reasons were increased β-hCG and detection of tubal rupture during follow-up. For 18.9% of methotrexate treatment it was not possible do determine success due to lack of follow-up. When assessing the frequency of the use of surgical access routes over the years, we observed a significant trend towards an increase in the use of laparoscopic access and a reduction in laparotomy (z = 2.09; p = 0.03) ( Figure 2 ).

**Fig. 3:**
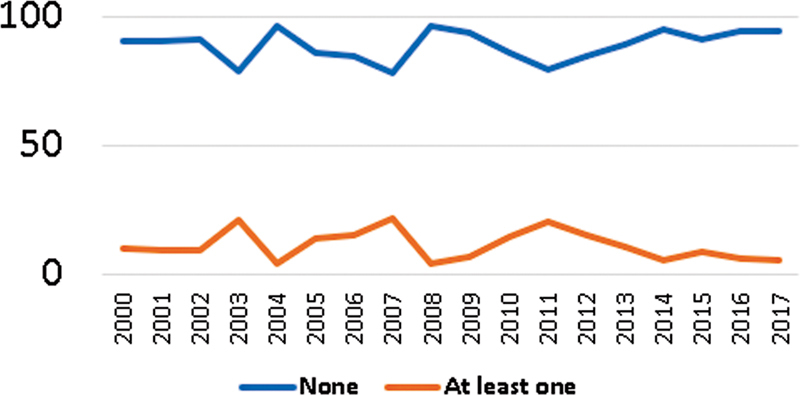
Severe complications between 2000 and 2017* Cochran-Armitage test: z = -0.95; p = 0.342

Of the women in our sample, 71 (10.55%) developed some type of severe complication associated with ectopic pregnancy. The most common complication was the need for blood transfusion (8.1%), followed by admission at the intensive care unit (4.3%). During the 17-year study period: only 6 (0.8%) of the women required reoperation, 5 (0.7%) underwent hysterectomy as a consequence of ectopic pregnancy, and no deaths due to ectopic pregnancy were registered. When assessing the frequency of severe complications over the years, we did not notice any significant difference (z = -0.95; p = 0.342) ( Figure 3 ).

We found that severe complications were significantly more common among white women (p = 0.01), who had a ruptured ectopic pregnancy (p <0,01), cases with a non-tubal location (p <0.01), those who did not present with vaginal bleeding (p < 0.01), with abdominal pain (p < 0.01), without previous ectopic pregnancy (p <0.01), those who had no history of abdominal surgery (p = 0.01), and nonsmokers (p = 0.02). Among the women who developed severe complications, the initial treatment was surgical in 90% (p < 0.01) and 29.4% underwent median laparotomy (p < 0.01) ( Table 4 ).

**Table 4:** Factors associated with severe complications in patients diagnosed with ectopic pregnancy (categorical variables)

Variables	Severe complication		n	p-value
	Yes (%)	No (%)		
Location				0.004 b
Tubal	85.92	95.35	635	
Nontubal	14.08	4.65	38	
Abdominal pain				< 0.001 a
Yes	83.10	62.17	432	
No	16.90	37.83	239	
Vaginal bleeding				< 0.001 a
Yes	43.66	64.17	416	
No	56.34	35.83	255	
Previous ectopic				0.002 a
Yes	2.82	17.11	105	
No	97.18	82.89	568	
**Previous laparotomy**				0.011 a
Yes	11.27	24.75	157	
No	88.73	75.25	516	
**Smoking**				0.029 a
Yes	15.63	28.52	156	
No	84.38	71.48	420	
**Skin color**				0.015 a
White	83.61	68.59	420	
Non-white	16.39	31.41	179	
**Ectopic pregnancy integrity**				< 0.001 a
Non-ruptured	27.94	63.23	399	
Ruptured	72.06	36.77	270	
**Type of treatment (first choice)**				< 0.001 a
Surgical	90.14	61.30	433	
Methotrexate	8.45	27.91	174	
Expectant	1.41	10.80	66	
**Surgical access route**				< 0.001 b
Laparoscopy	2.94	24.04	108	
Laparoscopy followed by Pfannenstiel	1.47	0.91	5	
Median laparotomy	29.41	11.11	69	
Pfannenstiel	66.18	63.95	327	
Median laparotomy	29.41	11.11	69	
Pfannenstiel	66.18	63.95	327	

a Chi-square test.

b Fisher's exact test.

As can be seen in Table 5 , the occurrence of severe complications was also correlated with some quantitative variables, such as higher levels of β-hCG (p < 0.01), larger diameter of ectopic pregnancy (p = 0.01), shorter stature (p = 0.049), higher gestational age determined by ultrasound (p = 0.019), and longer hospital stay (p < 0.01).

**Table 5:** Distribution of continuous variables according to the presence of severe complications after ectopic pregnancy

Variables	Severe complication		p-value*
	Yes	No	
	Mean ± SD	Mean ± SD	
Serum β-hCG at diagnosis (mIU/mL)	16,325 ± 24,529	5,300.9 ± 10,459	0.005
Gestational sac diameter (mm)	43.20 ± 21.40	36.73 ± 19.96	0.016
Methotrexate dose (mg)	93.50 ± 32.89	86.7 ± 21.76	0.921
Height (cm)	159.46 ± 7.50	161.11 ± 6.52	0.049
Body surface area (m ^2^ )	1.69 ± 0.19	1.69 ± 0.16	0.833
Gestational age at diagnosis (ultrasound-weeks)	9.91 ± 4.33	7.46 ± 2.06	0.019
Number of previous pregnancies	1.61 ± 1.52	1.72 ± 1.51	0.452
Number of previous cesarean sections	0.40 ± 0.75	0.43 ± 0.77	0.660
Length of hospital stay (days)	3.85 ± 2.23	3.14 ± 1.94	< 0.001

In the final statistical model, the prevalence of severe complications was found to be higher in women who: were diagnosed with a ruptured ectopic pregnancy at admission (PR = 2.97; 95% CI: 1.61–5.46) did not present with vaginal bleeding (PR = 2.45; 95% CI: 1.41–4.25), had never undergone laparotomy or laparoscopy (PR = 6.69; 95% CI: 1.62–27.53), had a non-tubal ectopic pregnancy (PR = 4.61; 95% CI: 1.98–10.74), and do not smoke (PR = 2.41; 95% CI: 1.08–5.36) ( Table 6 ).

**Table 6:** Variables associated with severe complications – Multiple Cox Regression (n = 548)

Variables	p-value	P.R.*	95% CI PR*
Ectopic pregnancy integrity at diagnosis			
Non-ruptured (ref)	−	1,00	−
Ruptured	<0,001	2,97	1,61–5,46
Vaginal bleeding			
Yes (ref)	−	1,00	−
No	0,002	2,45	1,41–4,25
Surgical history such as laparotomy or laparoscopy			
Yes (ref)	−	1,00	−
No	0,009	6,69	1,62–27,53
Smoking			
Yes	−	1.00	−
No	0.031	2.41	1.08–5.36
Ectopic pregnancýs location			
Tubal (ref)	−	1,00	−
Non-tubal	<0,001	4,61	1,98–10,74

## Discussion

Ectopic pregnancy has significant repercussions on women's health, in terms of morbidity and mortality, and there have been few studies evaluating the treatments and factors associated with a worse prognosis among them in Brazil. The main objective of this study was to compare the rates of methotrexate, surgical, and expectant management and to evaluate severe complications of ectopic pregnancy in a university hospital in the south eastern region of Brazil over a period of 17 years.

Some studies have suggested that the use of methotrexate in the clinical treatment of ectopic pregnancy, in cases that meet the eligibility criteria (gestational sac diameter < 4 cm, serum β-hCG ≤ 5,000 IU, absence of a fetal heartbeat, hemodynamic stability, and no contraindications), has the same success rate as surgical treatment. 8 In addition, in well-selected patients, treatment with methotrexate had a better cost-benefit ratio than surgery. 3 8 11 15 Because ectopic pregnancy is being diagnosed earlier and treatment protocols based on methotrexate have been developed, there is a trend toward an increase in the number of methotrexate treatments, in comparison with that of surgical treatments, in several countries. 3 9 11 16 17 18 In line with worldwide standards, the trends over the years at the university hospital studied were toward an increase in the rates of methotrexate treatment, possibly due to earlier diagnosis. Some studies point to an increase in expectant management rates in recent years, 18 which was not observed in our study. The inevitable question, however, is whether all health care facilities in Brazil show similar trends or whether it is a peculiarity of university, tertiary and private care centers.

In the present study, 40.3% of the patients were diagnosed with a ruptured ectopic pregnancy at admission. Among the surgical access routes available for the surgical treatment of ectopic pregnancy, laparoscopy has less morbidity than laparotomy, provided that a trained team is available. 1 Some studies have also suggested that patients undergoing laparoscopy require less blood transfusion and will have fewer pelvic adhesions than those who undergo laparotomy, which minimizes the impact on the reproductive future. 19 20 21 22 23 During the period of analysis, we observed a significant increase in the use of laparoscopy, which may be related to a greater availability of surgical instruments and a better adaptation of the team to the surgical technique. However, it is possible that the same does not happen in other health services in the country.

We found that 10.55% of the sample developed some type of serious complication associated with ectopic pregnancy. The most common complication was the need for blood transfusion, followed by admission to the intensive care unit. This frequency remained stable throughout the period of analysis, despite the increased use of methotrexate treatment and laparoscopy. The non-fatal complications of ectopic pregnancy are poorly studied. 24 Some observational studies have reported surgical complications in 23.4% of cases 25 and blood transfusion rates of 4.8% regardless of the type of treatment. 18

As for the factors that were most associated with the severity of the cases, we noticed that women who had a ruptured EP at admission, who did not have vaginal bleeding, had non-tubal EP, had never undergone laparotomy or laparoscopy, and who did not smoke had a higher prevalence of complications. Clearly, delayed diagnosis tends to have an impact on the evolution of the disease, increasing the risk of rupture prior to admission and of an unfavorable evolution. During the study period, there were 270 cases in which the ectopic pregnancy had already ruptured prior to diagnosis, accounting for 40.3% of all cases. Although we have the means of early diagnosis and clinical treatment in the hospital, we are also a reference for other cities in the region, from where we usually receive many cases at advanced evolution that do not warrant other ways of management than surgery.

It is possible that the absence of vaginal bleeding can decrease the chance of early diagnosis because health professionals are looking for the classic triad of positive β-hCG, abdominal pain, and vaginal bleeding. It is possible that women, who seek emergency care for abdominal pain, without vaginal bleeding, are misdiagnosed, and the diagnosis is only made during the second consultation. In addition, irregular or unexpected vaginal bleeding tends to be an early warning sign that prompts patients to seek immediate emergency care. Another hypothesis to explain this association is related to the notion of the evolutionary nature of pregnancy; that is, pregnancies in which there is a greater amount of trophoblastic tissue (i.e., those with longer evolution) will have an ascending curve and higher levels of β-hCG. Consequently, they will have higher levels of progesterone and less vaginal bleeding due to endometrial desquamation. 20 Ectopic pregnancy that does not present with vaginal bleeding tends to be characterized by delayed diagnosis with a potential for greater severity.

Non-tubal ectopic pregnancies also tend to be diagnosed later and present a greater degree of difficulty in the surgical approach. Unusual sites for trophoblast implantation include the cervix, cornual, and ovaries as well as abdominal scars from previous cesarean sections, the frequency of the latter being on the rise due to an increase in cesarean delivery. 25 26 27 When trophoblast implantation occurs in the uterine cornus and surgery is required, the rates of associated bleeding are often higher, due to the thickness of the myometrium in this region, together with the abundant vascularization resulting from trophoblastic implantation. 28 The difficulty in repairing it, associated with bleeding, can lead to an emergency hysterectomy. 29 30 The ovaries are irrigated by the ovarian artery, an arterial branch of the aorta. Therefore, in addition to the risk of oophorectomy and impaired reproductive future, 31 ovarian ectopic pregnancy carries a great risk of hemorrhage. When trophoblast implants in a cesarean scar, the risk of uterine rupture and shock is a considerable possibility, and this type of ectopic pregnancy is associated with placenta percreta in more advanced pregnancies. 32 Cervical ectopic pregnancy presents difficulties in surgical access due to the proximity of the uterine arteries and ureters, and can present with postoperative complications such as: hemorrhage, the need for hysterectomy, and urinary tract injury. 33 Abdominal pregnancy also presents serious risks as it can occur close to the liver, spleen, and intestinal loops, which evolve with difficulty in controlling hemorrhage and fecal peritonitis. 34 In our study, we observed an association between the absence of abdominal surgery, no smoking and a higher occurrence of serious complications. We are not aware of any study that has previously found similar associations, and we have no hypothesis that can explain these findings. Possibly, as several associations were made between variables, there may have been multiple comparison bias. Therefore, further studies are needed to assess these possible associations.

This study illustrates 17 years of monitoring cases of ectopic pregnancy in a university hospital, permitting not only the description of the variables related to the diagnosis and management of cases, but also the observation of trends. However, it has some limitations. Due to the retrospective characteristics and the cross-sectional analysis of the data, it was not possible to establish cause-and-effect relationships. Additionally, due to the large number of variables analyzed, there may have been multiple association biases. We believe, however, that the results are valid, since we analyzed a considerable number of cases over a long period of time, thereby contributing to the discussion and analysis of the management of ectopic pregnancy cases in Brazil.

## Conclusion

In conclusion, we observed that there was a change in the first treatment option for cases of ectopic pregnancy in the hospital during the period of analysis. There was a change in management of ectopic pregnancy with reduced surgeries and increased methotrexate treatment. This is possibly related to the development of treatment protocols based on methotrexate, in addition to the earlier diagnosis of the disease. We also observed an increase in the use of laparoscopy, which represents an improvement in the quality of care for women. Factors inherent to a disease that is more difficult to treat, such as non-tubal ectopic location together with conditions related to late diagnosis, are related to a higher frequency of serious complications. The results obtained may contribute to the reduction of maternal morbidity and mortality in our country and improve the quality of care for women.
